# EEG dynamical correlates of focal and diffuse causes of coma

**DOI:** 10.1186/s12883-017-0977-0

**Published:** 2017-11-15

**Authors:** MohammadMehdi Kafashan, Shoko Ryu, Mitchell J. Hargis, Osvaldo Laurido-Soto, Debra E. Roberts, Akshay Thontakudi, Lawrence Eisenman, Terrance T. Kummer, ShiNung Ching

**Affiliations:** 1 Department of Electrical and Systems Engineering, Washington University in St. Louis, 1 Brookings Dr. Campus Box 1042, St. Louis, MO 63130 USA; 2 Department of Neurology, Washington University School of Medicine, 660 S Euclid Ave. Campus Box 8111, St. Louis, MO 63110 USA; 3 Division of Biology and Biomedical Science, Washington University in St. Louis, St. Louis, MO 63110 USA; 4 Present Address: Harvard Medical School, Boston, USA; 5 Present Address: Department of Neurology, Novant Health Forsyth Medical Center, Winston-Salem, USA; 6 Present Address: Department of Neurology, University of Rochester, Rochester, USA

**Keywords:** Coma, Classification, Electroencephalogram, Depressed level of consciousness

## Abstract

**Background:**

Rapidly determining the causes of a depressed level of consciousness (DLOC) including coma is a common clinical challenge. Quantitative analysis of the electroencephalogram (EEG) has the potential to improve DLOC assessment by providing readily deployable, temporally detailed characterization of brain activity in such patients. While used commonly for seizure detection, EEG-based assessment of DLOC etiology is less well-established. As a first step towards etiological diagnosis, we sought to distinguish focal and diffuse causes of DLOC through assessment of temporal dynamics within EEG signals.

**Methods:**

We retrospectively analyzed EEG recordings from 40 patients with DLOC with consensus focal or diffuse culprit pathology. For each recording, we performed a suite of time-series analyses, then used a statistical framework to identify which analyses (features) could be used to distinguish between focal and diffuse cases.

**Results:**

Using cross-validation approaches, we identified several spectral and non-spectral EEG features that were significantly different between DLOC patients with focal vs. diffuse etiologies, enabling EEG-based classification with an accuracy of 76%.

**Conclusions:**

Our findings suggest that DLOC due to focal vs. diffuse injuries differ along several electrophysiological parameters. These results may form the basis of future classification strategies for DLOC and coma that are more etiologically-specific and therefore therapeutically-relevant.

**Electronic supplementary material:**

The online version of this article (doi: 10.1186/s12883-017-0977-0) contains supplementary material, which is available to authorized users.

## Background

A depressed level of consciousness (DLOC) is a near universal result of acute severe brain injury, and disorders of consciousness are among the most feared long-term sequelae of such injuries. Coma, a state of complete loss of spontaneous or stimulus-induced arousal, is the most severe form, but all forms of DLOC have substantial impacts on patient outcomes [1, 2]. A DLOC can result from diffuse brain injuries, or from focal insults to brain regions with widespread projections that secondarily induce global alterations in cerebral function [3]. For example, diffuse axonal injury may induce a diffuse DLOC through widespread cortical deafferentation, while a small brainstem hemorrhage may induce a focal DLOC via an injury to the ascending reticular activating system. Formulating an accurate differential is crucial to the clinical management of patients with DLOC, as diagnoses drive the approach to treatment and prognosis [2–4]. Diagnostic formulation often begins with distinguishing between focal and diffuse etiologies.

In some cases a careful history, paired with a basic laboratory workup and screening neuroimaging tests, are all that are required to determine the cause of coma or other DLOC. Often, however, these standard assessments prove inadequate to determine DLOC etiology during acute, therapeutically-relevant windows. There are several common scenarios in which such ambiguity exists: A patient may have a DLOC that exceeds expectations from modest structural brain injury evident on imaging; or a patient’s DLOC may result from a focal process that, due to its nature, acuity, location, or size, is not apparent on screening imaging studies.

More specialized testing, such as expanded laboratory assessments, specialized neuroimaging studies, and invasive procedures, may help to establish a diagnosis. These tests, however, carry risk and expense and are only useful in restricted circumstances. Similarly, highly specialized interventions including specific medications and even surgical procedures are effective in some cases, but are rarely used empirically. A non-invasive, bedside screening test that can help classify DLOC acutely could be of significant utility in guiding both advanced diagnostic strategies and, ultimately, management approaches [5, 6]. Although the differentiation of focal from diffuse DLOC may not be clinically-actionable on its own, it may help distinguish between more specific diagnoses that are, or suggest a more targeted work-up. A strong suggestion of a focal etiology in the absence of initial imaging findings, for example, might prompt more advanced neuroimaging. Similarly, a strong suggestion of a diffuse etiology, even in the presence of distracting structural brain lesions, might prompt a more extensive toxic-metabolic work-up, or a more aggressive correction of known toxic-metabolic abnormalities.

All DLOC, and in particular coma, are characterized by pathological alterations in brain electrical activity [7]. These electrical alterations may provide valuable etiological insight. Consistent with this, in addition to its well-established role in seizure detection, the EEG has been shown to have utility in the monitoring of non-epileptic, large-scale alterations of neurological function. Examples include EEG monitoring of delirium [8, 9], burst suppression [10, 11], and cerebral ischemia [12–14]. Thus, EEG can provide non-invasive, highly temporally-resolved data at the bedside on both structural and non-structural brain injury that may not be apparent on screening neuroimaging studies.

Visual inspection of raw EEG data requires advanced training and cannot easily capture the full complexity of electrical dynamics that are potentially encoded in the EEG signal. In contrast, quantitative EEG methods use computer-assisted analysis of EEG patterns to derive quantitative metrics that are not immediately apparent upon review of raw EEG data. The use of quantitative EEG analysis in the clinical setting has seen significant recent growth, particularly in the domains of sleep [15], epilepsy [16], and general anesthesia [17]. In these scenarios, progress has been made towards translational applications including seizure detection [16, 18], classification of sleep stages [19, 20], and quantification of depth of anesthesia [21, 22].

While a DLOC is expected to entail widespread network dysfunction regardless of injury type, secondary network dysfunction resulting from focal injuries may exhibit temporal or other EEG features distinct from those of primarily diffuse injuries. The goal of this study is to evaluate quantitative EEG analysis for classifying focal and diffuse DLOC [23, 24] with a particular focus on the *temporal* dynamics of the EEG. In other words, do focal injuries give rise to different temporal dynamics as compared to diffuse injuries? This approach contrasts spatial analyses that overtly characterize inter-region relationships (e.g., inter-hemispheric symmetry) with respect to a particular temporal signature (see also Discussion). If successful, such strategies may eventually become applicable to more specific, clinically-actionable DLOC etiological subtypes.

Common treatments of temporal dynamics in EEG involve spectral analysis, which decomposes a given signal into constituent frequencies [25], typically aggregated into the standard EEG ‘bands’ (i.e., alpha, delta, etc.) [26]. In this context, severe brain injuries and DLOC are classically associated with concentration of EEG power into low frequencies (<1 Hz) [27]. However, while approaches based on spectral analysis are commonplace, this form of analysis only captures sinusoidal harmonic structure in the underlying signal. Other forms of spatiotemporal time series analysis, such as measures of signal entropy and complexity, are available that may complement and augment spectral methods [28], and have been applied to EEG data from limited cohorts of patients with DLOC [29, 30]. We investigated these and other temporal markers to determine their potential for classifying focal and diffuse DLOC.

## Methods

### Study population and data collected

We retrospectively collected EEG data, EEG reports, and complete medical records from 62 patients who underwent EEG for routine monitoring purposes related to a diagnosis of coma or less-severe DLOC, which we define as a Glasgow Coma Scale (GCS) ≤ 9 at the time of EEG, in the Neurological and Neurosurgical Intensive Care Unit at Barnes-Jewish Hospital and Washington University School of Medicine (St. Louis, MO, USA). GCS was inferred for intubated patients [31]. Table 1 summarizes the study population and clinical determinations. Additional file 1: Table S1.

**Table 1:** Summary of study population

Classification	Diffuse	Focal
	*N* = 19 (47%)	*N* = 21 (53%)
Male	6 (32%)	12 (57%)
Female	13 (68%)	9 (43%)
Age	58.32 (23, 90)	58.42 (18, 87)
GCS at time of EEG	5.74 (3, 8)	5.6 (3, 9)
Injuries Observed		
Vascular	7	14
Diffuse structural	3	0
Brainstem lesion	0	6
Traumatic	2	1
Toxic/Metabolic	7	0

gives further clinical features for each subject included in this study. There were 62 patients considered and 70 total EEG studies (6 patients underwent EEG monitoring twice and one patient underwent EEG monitoring three times). In all cases EEG was performed for the detection of non-convulsive seizures in patients with otherwise inadequately explained DLOC. Only cases in which seizures were not detected at any point in the hospitalization were analyzed. For each of the 62 patients, two neurointensivists (TTK and either DER or MJH and OLS) examined all diagnostic data available including imaging to assign a focal or diffuse classification. Importantly, these assessments benefited from diagnostic data not available to the team at the time of the initial EEG. Thus in most cases we were able to determine DLOC etiology to a reasonable degree of clinical certainty despite the diagnostic ambiguity that resulted in EEG testing early on. Imaging data was given the greatest weight in etiological determinations. Evidence of herniation or direct injury to brainstem reticular activating structures resulted in assignment to a focal etiology. Less severe structural lesions were interpreted in the context of historical data and coexisting toxic-metabolic influences to determine the etiology of the DLOC. Cases were included in the analysis only when the ultimate etiology (focal vs. diffuse) was apparent from clinical data. In cases of disagreement, the case was re-reviewed and discussed until a consensus was reached (most such cases classified as indeterminate). In total 40 subjects (21 focal and 19 diffuse) were used in the analysis (23 focal and 21 diffuse EEGs, with total 44 studies analyzed). Twenty eight of 40 subjects (70%) had some evidence of more than one potential DLOC contributor, but in all included cases secondary causes were felt to be minor. To examine the utility of traditional clinical EEG metrics, the clinical EEG reports were separately scrutinized to identify reported features that could assist in the classification of cases as focal or diffuse in etiology. Specifically, any focal or lateralized abnormalities in the report were flagged as supportive of a focal as opposed to diffuse etiology. All studies were conducted with approval from the institutional review board at Washington University in St. Louis.

### EEG sampling and parsing

Recordings are collected at a sampling frequency of either 250 or 500 Hz using the standard 10–20 system of electrode placement. The 500 Hz data were downsampled to 250 Hz prior to analysis. In our analyses, we used a bipolar montage with 18 bipolar channels (FP1-F7, F7-T7, T7-P7, P7-O1, Fp1-F3, F3-C3, C3-P3, P3-O1, Fz-Cz, Cz-Pz, Fp2-F4, F4-C4, C4-P4, P4-O2, Fp2-F8, F8-T8, T8-P8, and P8-O2). Records were visually analyzed for quality control, with sections of the record containing large-amplitude artifacts excluded from analysis. Each bipolar channel was normalized to zero-mean, unit variance. They were then filtered using a 10th order Chebyshev Type I lowpass filter with cutoff frequency of 50 Hz before further analysis.

### Feature extraction and classification

#### Feature extraction

We considered the 25 features listed in Table 2 for discrimination of focal and diffuse DLOC. These features are related to the dynamical (including spectral) properties of time-series data. Secondary statistics (e.g., higher order moments) of the features were not considered in this analysis. All signal processing and feature extractions were performed in MATLAB (Natick, MA), and feature selection and evaluation of classifiers were computed in R (version 3.1.2).

**Table 2:** List of features. List of 25 features extracted from EEG data

Feature ID	Description
1–2	Maximum, minimum eigenvalues of the estimated **A** matrix from MVAR fitting of EEG data with unit order; see Eq. (1)
3	Number of absolute eigenvalues of matrix **A** larger than Threshold = 0.95; see Eq. (1)
4–6	Statistical properties: variance, skewness, and kurtosis
7–11	Power in the delta, theta, alpha, beta, and gamma bands
12	Ratio of power in beta and gamma bands to total power
13	Ratio of power in delta and theta bands to total power
14	Hurst exponent [33]
15	Hjorth parameters [33]
16–19	Equidistant mutual information, Equiprobable mutual information, and the first minimums of both types of mutual information [33]
20	Bicorrelation
21	Median frequency [33]
22–24	Spearman autocorrelation, Pearson autocorrelation, and partial autocorrelation
25	Composite permutation entropy index (CPEI) [62]

#### Definition of trials and analysis epochs

**Fig. 1:**
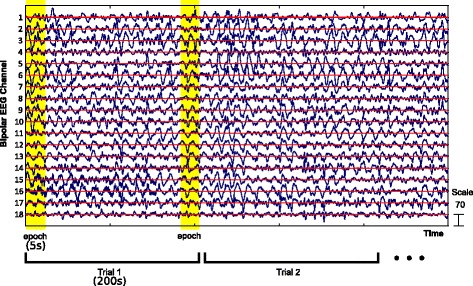
Schematic illustrating sliding window to define epoch and trial in EGG data with bipolar montage. The EEG channel number on the vertical axes are ordered as: FP1-F7, F7-T7, T7-P7, P7-O1, Fp1-F3, F3-C3, C3-P3, P3-O1, Fz-Cz, Cz-Pz, Fp2-F4, F4-C4, C4-P4, P4-O2, Fp2-F8, F8-T8, T8-P8, P8-O2

We divided each patient’s bipolar montaged EEG data into separate, non-overlapping trials for the purpose of analysis (Fig. 1). Dividing the EEG data into trials results in multiple predictions for each subject, thus yielding an empirical probability of focal/diffuse for each subject. Each trial was further subdivided for analysis purposes into epochs (Fig. 1). Specifically, one 25-dimensional feature vector for each trial by averaging feature vectors over all epochs of that trial. In our analysis, we considered trial lengths of 200 s, with epoch lengths of 5 s (i.e., 40 epochs per trial), except as noted when evaluating the robustness of our obtained features. It is important to note that all the trials for a given subject were allocated to either the training or testing set, thus training and testing sets are fully independent (i.e., no subject contributed trials to both the training and testing sets; see also below).

#### Classification and training/testing separation

We specified a support vector machine (SVM) to function as a binary classifier to discern a patient’s DLOC etiology (i.e., focal or diffuse). The SVM approach uses a portion of data as support vectors to create a decision boundary (i.e., threshold) [32, 33]. To train the classifier, we applied principal component analysis (PCA) to the primary feature vectors over trials. Only the first 20 most important principal components (PCs) of feature vectors were kept. The first 20 PCs explain more than 98% of the variance in the original feature vectors (data not shown). We used the *train.R* routine in the Caret toolbox [34], implemented in the R programming language, to rank the features/PCs by importance by evaluating a family of linear vector quantization (LVQ) models. A 10-fold cross validation is used within this feature selection step which is resampled 50 times. We then selected a set of predictive PCs based on their importance (i.e., quantified in terms of their ensuing LVQ classification performance) to train a SVM with a linear kernel according to different clinically-adjudicated DLOC etiologies. Within this cross-validation paradigm, the importance of a feature/PC was obtained as a normalized quantity that characterizes the relative improvement in the region under the receiver operating characteristic (provided by the feature in question). In other words, the extent to which that feature improves accuracy within the cross-validation paradigm.

All classification analysis was performed using strictly independent training and testing sets. Two testing paradigms were considered. In the first paradigm, we partitioned the data into two groups: two-thirds of the total subjects were selected randomly and defined as the training set and one-third of subjects were defined as the testing set. This process was repeated within a cross-validation paradigm in order to evaluate average classification performance. In the second paradigm, we withheld 14 patients (1/3 of the data) as a dedicated test set, and trained strictly on the remaining 2/3 of the patients (i.e., one-time training and testing, with no re-sampling and averaging).

#### Evaluation of classifier performance

Classification performance was evaluated in two ways: (i) Hard accuracy, wherein each trial from a subject was independently classified, with the overall classification being made on the basis of the majority of trials; (ii) Soft accuracy, wherein each trial was independently classified with no overall classification rendered.

#### Multivariate autoregressive model of EEG data

Features 1–3 in Table 2 use a multivariate autoregressive model wherein the (multivariate) EEG signal is modeled as a linear sum of previous samples. For a multivariate N-channel process**x**(*t*) = [**x**_1_(*t*), **x**_2_(*t*), ⋯, **x**_*N*_(*t*)]^*T*^, a Multivariate Autoregressive (MVAR) Model of order p takes the following form:

1 $$ \mathbf{x}(t)=\sum \limits_{k=1}^p{\mathbf{A}}_k\mathbf{x}\left(t-k\right)+\mathbf{w}(t), $$

where **w**(*t*) ∈ **ℝ** ^*N*^are additive noise vectors (innovation process) and **A**_*k*_ ∈ **ℝ** ^*N* × *N*^are the MVAR model coefficients. Here, we used a standard Least-Squares approach to implement the model fit [35].

#### Statistical evaluation

We used a two-sample t-test to compare feature distributions. Our goal was to generate hypotheses regarding which of the screened features were informative with respect to the considered coma subtypes (focal and diffuse). Since the PCs of the primary features are uncorrelated (see Results), we compared subtype distributions of each PC independently to a nominal significance level of *p* = *0.05*.

## Results

### Several time-series metrics, including non-spectral features, discriminate focal from diffuse DLOC

#### Correlation of primary features

**Fig. 2:**
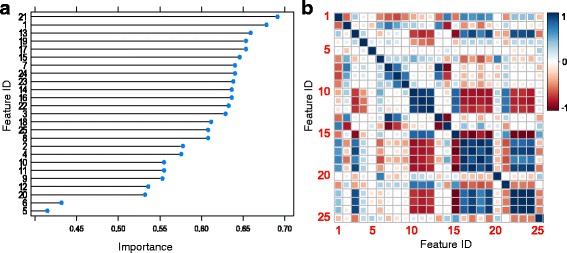
**a** Ranking of features by importance (see Methods). **b** Correlation between features, noting in particular substantial redundancy in the entropic features 15–24

**Fig. 3:**
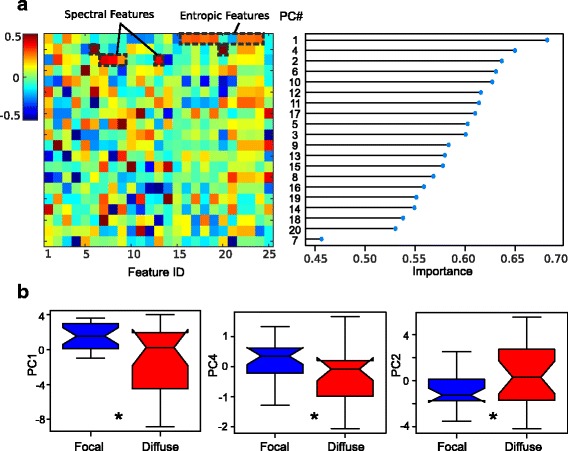
**a** (left) Principal component decomposition of primary features. Each row in the matrix depicts the composition of a PC. The colorbar references the weight of each primary feature to the respective PCs. (right) Rows are ranked according to PC importance. **b** Box plots comparing the distributions of PC1 (entropic features), PC4 (kurtosis, bicorrelation) and PC2 (delta, alpha, theta, delta/theta ratio) for focal and diffuse cases

We first screened and ranked the importance of the primary features, i.e., without applying PCA (Fig. 2a) using testing paradigm 1, i.e., with resampling and cross-validation. As illustrated in Fig. 2b, these primary features exhibit substantial correlation, particularly in the entropic features (i.e., features 15–24 in Table 2). This observation indicates a degree of redundancy in the discriminative power of these features. We also note substantial anti-correlation between spectral and entropic features (i.e., 10–13 and 15–24). Recall that the importance of a feature measures its relative ability to improve classification performance, where an importance of 1 means that the feature in question can alone lead to perfect accuracy. Thus, we transformed the primary features into their uncorrelated principal components, then ranked these PCs (Fig. 3a).

#### Dichotomy of entropic and spectral features

We observed that the most informative PCs dichotomized into two categories (Fig. 3a): (i) components comprised mostly of entropic time series analyses (i.e., PC1) and higher order statistical signal properties (PC4); and (ii) components comprised mostly of spectral analyses (i.e., band-limited power, (delta, alpha, theta) and delta/theta ratio PC2). This observation is in agreement with the anti-correlation between these categories observed in Fig. 2b. Subsequent PCs, while informative, are comprised of a more random combination of analyses. The composition of these PCs is notable since by definition these components are uncorrelated, meaning that they provide distinct information regarding DLOC subtype. It is interesting to observe that the least informative PC (PC7) has a strong contribution from the median frequency, so that this feature is not particularly useful for discriminating focal and diffuse etiologies.

#### Variability in diffuse cases

Figure 3b compares the distribution of the three most informative PCs (distributions of focal/diffuse cases are most significantly different). We observed a positive contribution of entropic features (PC1) and higher order statistical signal properties (PC4) to focal cases, versus PC2 which has a positive contribution for diffuse cases. Further, we observed greater variance associated with diffuse cases, which may be suggestive of heterogeneity across channels or trials in these cases (see Discussion). Changing the feature epoch lengths (1, 10, 20 s) had no qualitative effect on the overall results (data not shown).

### Focal and diffuse DLOC are classified using a limited number of PCA features

We next designed a classifier for focal versus diffuse DLOC on the basis of first the primary features and then the PCs. Table 3 shows the classifier performance using the primary features only (i.e., without applying PCA) for different epoch lengths and number of epochs in a trial. In this analysis, the classifier is trained based on the 10 most informative features on two thirds of the subjects (27 subjects), and then tested on the remaining patients (13 subjects). We performed training and testing for 500 repetitions (each repetition, subjects in the training set are totally separate from subjects in testing set). The values reported in this table are averaged over repetitions. The classifier exhibits higher sensitivity (true positives; here ‘positive’ is defined as a focal etiology) compare to specificity (true negatives, i.e., diffuse trials that were classified as diffuse). In this analysis, false positives (misclassification of diffuse as focal etiology) constitute the predominant source of inaccuracy. This Table shows that classifying on the basis of primary features alone is inadequate since performance is not significantly above chance, and particularly poor in correctly identifying diffuse cases.

**Table 3:** Averaged classification performance before applying PCA on initial features

Epoch length	Number of epochs in a trial	Accuracy (Hard)	Accuracy (Soft)	Specificity	Sensitivity
1 s	10	0.51	0.52	0.42	0.66
	20	0.53	0.54	0.45	0.68
	40	0.53	0.53	0.41	0.69
	100	0.53	0.53	0.41	0.70
	All	0.57	0.57	0.49	0.69
5 s	10	0.50	0.49	0.44	0.67
	20	0.50	0.50	0.41	0.68
	40	0.52	0.51	0.44	0.67
	100	0.58	0.54	0.51	0.66
	All	0.57	0.57	0.46	0.72
20 s	10	0.51	0.51	0.41	0.69
	20	0.52	0.51	0.38	0.70
	40	0.53	0.53	0.51	0.64
	All	0.59	0.59	0.46	0.77

Classification results are for different epoch length (1 s, 5 s, and 20s) and different number of epochs in a trial (10, 20, 40, 100, and all epochs). All the results are averaged classifier performance over 500 random training and testing sets. In each realization, the subjects in training and testing sets are different

Thus, we redesigned the classifier using the PCs, after which the performance reported in Table 4 was obtained. Only the first six most informative PCs are used for classification. Working with the PCs results in substantial improvement to the overall classifier performance. Specifically, it can be seen in this table that the false positive rate is improved on average by 0.14, and average performance approaches 68% for many parameterizations.

**Table 4:** Averaged classification performance after applying PCA on initial features

Epoch length	Number of epochs in a trial	Accuracy (Hard)	Accuracy (Soft)	Specificity	Sensitivity
1 s	10	0.63	0.63	0.57	0.68
	20	0.64	0.62	0.59	0.68
	40	0.65	0.62	0.59	0.68
	100	0.65	0.63	0.59	0.69
	All	0.62	0.62	0.55	0.67
5 s	10	0.63	0.61	0.57	0.67
	20	0.65	0.64	0.60	0.68
	40	0.65	0.62	0.60	0.69
	100	0.68	0.65	0.57	0.71
	All	0.65	0.62	0.61	0.66
20 s	10	0.63	0.59	0.55	0.66
	20	0.63	0.59	0.56	0.65
	40	0.64	0.61	0.59	0.68
	All	0.64	0.60	0.61	0.67

Classification results are for different epoch length (1 s, 5 s, and 20s) and different number of epochs in a trial (10, 20, 40, 100, and all epochs). All the results are averaged classifier performance over 500 random training and testing sets. In each realization, the subjects in training and testing sets are different

Finally, we performed a final test of the classifier by withholding 13 subjects as a dedicated testing set, and trained the classifier on only the remaining cases. That is, we trained a classifier on 2/3 of the data, then evaluated it on a separate, withheld patient cohort (paradigm 2). Table 5 reports the classification performance in this scenario, where performance approaches 76% with high sensitivity, but only moderate specificity. It is important to emphasize that within these classification regimes, the feature extraction step is performed on only the (independent) training set.

**Table 5:** Classification performance after applying PCA on initial features with first 13 subjects as testing set

Epoch length	Number of epochs in a trial	Accuracy (Hard)	Accuracy (Soft)	Specificity	Sensitivity
1 s	10	0.69	0.69	0.66	0.71
	20	0.76	0.68	0.65	0.69
	40	0.69	0.71	0.66	0.73
	100	0.69	0.69	0.66	0.73
	All	0.69	0.67	0.52	0.77
5 s	10	0.76	0.69	0.62	0.73
	20	0.69	0.71	0.70	0.73
	40	0.76	0.70	0.66	0.71
	100	0.69	0.69	0.66	0.73
	All	0.61	0.61	0.52	0.73
20 s	10	0.61	0.61	0.51	0.71
	20	0.69	0.65	0.63	0.69
	40	0.61	0.67	0.66	0.71
	All	0.61	0.61	0.62	0.60

Classification results are for different epoch length (1 s, 5 s, and 20s) and different number of epochs in a trial (10, 20, 40, 100, and all epochs)

### Clinical EEG interpretation

Lastly, we examined the EEG reports for our study population to determine whether a similar classification performance could be achieved from a more straightforward clinical decision process. Specifically, we catalogued observations of focal or lateralized abnormalities in the EEG reports (lateralized or otherwise focal slowing or epileptiform discharges), an indication of a spatially local electrophysiological phenomenon correlating with focal injury. Of the 21 patients with focal injury in our study, a report of focal/lateralized slowing was only present in seven instances (i.e., corresponding to a sensitivity of 7/21 = 0.33), demonstrating that the clinical EEG report was a poor indicator of focal etiology. Generalized slowing was observed in all study patients.

## Discussion

### Disambiguating focal and diffuse DLOC etiologies using EEG time series analysis

Our results demonstrate proof-of-concept for EEG-based segregation of focal versus diffuse DLOC sub-types based on time-series analysis and support-vector machine classification. We evaluated the performance of this system by segmenting our data into separate training and testing sets and then using cross-validation to minimize model overfitting. The results demonstrate performance up to 76% accuracy in focal identification, but less robust performance in detecting diffuse cases. In many cases, including all those studied for this report, the ultimate cause of the DLOC only becomes clear in retrospect, hence the clinical decision to order an EEG to rule out seizures as a contributor to a DLOC. Thus even in cases where a clinical diagnosis can eventually be made, an EEG-based diagnostic method could assist with the timely delivery of care.

The design of our classifier reveals that potentially clinically-useful information regarding DLOC subtype may be embedded in both spectral and non-spectral features of the EEG signals of patients with DLOC. Our results further suggest that spectral analysis alone (e.g., band-limited power) may not capture all clinically-meaningful aspects of the underlying neuronal dynamics.

Other potentially useful approaches for detecting focal versus diffuse pathology from the EEG include the brain symmetry index (BSI), which examines inter-hemispheric symmetries in the power-spectral density (i.e., band limited power) [36, 37]. This method has been used in the detection of focal seizures and hemispheric strokes [38, 39]. EEG-based synchronization indices, also derived from the power-spectrum, have also been suggested as a means of detecting diffuse electrophysiological phenomena [40]. It stands to reason that such methods may be sensitive to injury focality, though to our knowledge none of these methods has been used in the context of DLOC or coma. Moreover, while approaches that focus on the spatial distribution of EEG power may be informative, we chose here to focus our attention on the EEG in terms of temporal dynamics only since we were interested in whether focal and diffuse injuring give rise to differing temporal patterns, without overt regard for their spatial distribution. Thus, our results are likely complementary to approaches such as the BSI and comparing the performance of the classifier reported herein with these indices, separately and in combination, is an important future goal. The incorporation of active stimuli to assess EEG reactivity may also provide additional information about DLOC [41, 42], and is a further target of future work.

Significant effort has been directed at the EEG-based analysis of chronic DLOC in rehabilitative settings, such as minimally conscious and persistent vegetative states [43, 44], wherein a large number of EEG analyses have been screened for their potential to disambiguate these subtypes [45]. While these studies have yielded insights into the mechanisms of these conditions, the analyses rely on high-density research grade EEG/MEG instrumentation [43, 46] that is generally unavailable in the acute setting, wherein electrode spatial density and placement precision is limited. It furthermore remains unclear whether insights gleaned from these studies will prove informative in the acute setting, though a recent study did identify several acute electrophysiological correlates of outcome in coma [47], demonstrating the prognostic potential of EEG-based approaches.

### Limitations

The main limitation in the development of our algorithm is the lack of a true gold standard upon which to train our classifier. Our cases were independently diagnosed by at least two neurointensivists on the basis of all available retrospective data, including neuroimaging, and only cases in which both felt a clear diagnosis was evident were included.

In this retrospective study we relied on the GCS for identification of patients with DLOC. The GCS is an imperfect measure of DLOC: its measurement is incomplete in intubated patients and many features that are likely to be clinically significant are not assessed. Several other metrics such as the Full Outline of UnResponsiveness (FOUR) score [48] and the JFK Coma Recover Scale, Revised [49] are likely to provide superior differentiation of patients with DLOC. Unfortunately our data did not permit such assessments retrospectively, though a prospective trial is underway including assessment of these metrics in addition to GCS.

A common challenge in the paradigm we pursued here is ensuring independent validation of classifier performance. We used two separate validation paradigms to separate training and testing sets (see Methods). Ongoing studies will test the performance of this classifier on additional independent, prospective cohorts of DLOC patients with clinically-obvious focal or diffuse DLOCs for whom EEG would not otherwise be clinically-indicated.

A drawback of our framework is that direct mechanistic interpretation from descriptive time-series analysis is lacking. The support vector machine approach aggregates all of the data/features (because the best predictors are combinations of the primary features), and then generates a set of predictors that may or may not be overtly linked to an underlying circuit mechanism. However, the decomposition of our PCs into distinct, uncorrelated feature sets (e.g., signal entropy and band-limited power) is suggestive of systematic circuit-level disruption in these patients.

Lastly, this analysis does not include specific steps to manage confounds introduced by the administration of medications such as antiepileptic drugs, including benzodiazepines, on the brain’s electrical activity. It is well-established that these medications, among other factors such as sleep [50, 51], can lead to confounding effects on the EEG. In the absence of a much larger trial matched for specific agents or classes of agents, it will be challenging to fully understand the impact of such confounds. It is likely that the confounding effects of these drugs constitute a substantial source of classification error in our dataset. In a future prospective trial it may be feasible to more specifically characterize the effect of classes of medications on the discriminatory power of our classification scheme in individual subjects.

### Design considerations

The feature selection framework reported above requires no manual specification of thresholds or other detection rules. The only user-specified design parameter is a desired confidence interval [52]. The method can be applied to any number of EEG channels. However, it should be noted that a pervasive challenge with any clinical EEG recording is overall signal quality and presence of artifacts (e.g., due to patient motion), which is expected to be compounded with added channels.

It is important to note that with this feature selection scheme, any change to the design parameters may lead to a different set of PCA features being chosen. Nevertheless, we found that across a range of design parameterizations (e.g., changing the trial length from 5 min to 10 min) the set of best predictors was largely comprised of the same descriptive time-series statistics. Thus it seems likely that these particular PCA features are robustly informative with respect to the two DLOC variants under investigation.

## Conclusions

The use of automatic classifiers in EEG is most well-developed in the detection of seizures [53–60]. A host of additional potential application domains have been considered, however, especially in the development of brain-machine interface technology [61]. Our results demonstrate the potential of using such techniques to assist in the diagnosis of DLOC in the acute setting. Moreover, the methods and algorithms used in our study run in minutes on standard hardware and, thus, could potentially enable real-time assessment of the EEG. If they are further validated on larger patient cohorts, they may form an important component of the overall assessment of acute DLOC.

## Additional file

Additional file 1: Table S1. Additional details regarding study population. (DOCX 12 kb)

## Abbreviations

BSI: Brain symmetry index
DLOC: Depressed level of consciousness
EEG: Electroencephalogram
FOUR: Full outline of unresponsiveness
GCS: Glasgow coma scale
LVQ: Linear vector quantization
MEG: Magnetoencephalogram
PC: Principal component
PCA: Principal components analysis
SVM: Support vector machine

## References

[1] Godbolt AK (2013). Disorders of consciousness after severe traumatic brain injury: a Swedish–Icelandic study of incidence, outcomes and implications for optimizing care pathways. J Rehabil Med.

[2] Whyte J (2013). Functional outcomes in traumatic disorders of consciousness: 5-year outcomes from the National Institute on Disability and Rehabilitation Research traumatic brain injury model systems. Arch Phys Med Rehabil.

[3] Giacino JT (2014). Disorders of consciousness after acquired brain injury: the state of the science. Nat Rev Neurol.

[4] Nakase-Richardson R (2012). Longitudinal outcome of patients with disordered consciousness in the NIDRR TBI model systems programs. J Neurotrauma.

[5] Laureys S, Boly M (2008). The changing spectrum of coma. Nat Clin Pract Neurol.

[6] Owen AM, Schiff ND, Laureys S (2009). A new era of coma and consciousness science. Prog Brain Res.

[7] Young GB (2000). The EEG in coma. J Clin Neurophysiol.

[8] van der Kooi AW (2015). Delirium detection using EEG: what and how to measure. Chest.

[9] Jacobson S, Jerrier H (2000). EEG in delirium. Semin Clin Neuropsychiatry.

[10] Westover MB (2013). Real-time segmentation and tracking of brain metabolic state in ICU EEG recordings of burst suppression. Conf Proc IEEE Eng Med Biol Soc.

[11] Ching S (2013). Real-time closed-loop control in a rodent model of medically induced coma using burst suppression. Anesthesiology.

[12] Claassen J (2004). Quantitative continuous EEG for detecting delayed cerebral ischemia in patients with poor-grade subarachnoid hemorrhage. Clin Neurophysiol.

[13] Jordan KG (2004). Emergency EEG and continuous EEG monitoring in acute ischemic stroke. J Clin Neurophysiol.

[14] Labar DR (1991). Quantitative EEG monitoring for patients with subarachnoid hemorrhage. Electroencephalogr Clin Neurophysiol.

[15] Şen B (2014). A comparative study on classification of sleep stage based on EEG signals using feature selection and classification algorithms. J Medical Syst.

[16] Claassen J (2004). Detection of electrographic seizures with continuous EEG monitoring in critically ill patients. Neurology.

[17] Brown EN, Lydic R, Schiff ND (2010). General anesthesia, sleep, and coma. N Engl J Med.

[18] Khan YU, Gotman J (2003). Wavelet based automatic seizure detection in intracerebral electroencephalogram. Clin Neurophysiol.

[19] Smith JR (1975). Detection of human sleep EEG waveforms. Electroencephalogr Clin Neurophysiol.

[20] Smith JR, Karacan I (1971). EEG sleep stage scoring by an automatic hybrid system. Electroencephalogr Clin Neurophysiol.

[21] Katoh T, Suzuki A, Ikeda K (1998). Electroencephalographic derivatives as a tool for predicting the depth of sedation and anesthesia induced by sevoflurane. Anesthesiology.

[22] Zhang X-S, Roy RJ, Jensen EW (2001). EEG complexity as a measure of depth of anesthesia for patients. Biomed Eng IEEE Trans On.

[23] Laureys S, Schiff ND (2012). Coma and consciousness: paradigms (re) framed by neuroimaging. NeuroImage.

[24] Schiff ND, Nauvel T, Victor JD (2014). Large-scale brain dynamics in disorders of consciousness. Curr Opin Neurobiol.

[25] Schomer DL, da Silva FL. Niedermeyer's electroencephalography: basic principles, clinical applications, and related fields. Philadelphia: Wolters Kluwer Health; 2012.

[26] Lehembre R (2012). Electrophysiological investigations of brain function in coma, vegetative and minimally conscious patients. Arch Ital Biol.

[27] Plum F, Posner JB (1982). The diagnosis of stupor and coma.

[28] Stam CJ (2005). Nonlinear dynamical analysis of EEG and MEG: review of an emerging field. Clin Neurophysiol.

[29] Chan HL, Lin MA, Fang SC (2004). Linear and nonlinear analysis of electroencephalogram of the coma. Conf Proc IEEE Eng Med Biol Soc.

[30] Gosseries O (2011). Automated EEG entropy measurements in coma, vegetative state/unresponsive wakefulness syndrome and minimally conscious state. Funct Neurol.

[31] Meredith W (1998). The conundrum of the Glasgow coma scale in intubated patients: a linear regression prediction of the Glasgow verbal score from the Glasgow eye and motor scores. J Trauma Acute Care Surg.

[32] Pereira F, Mitchell T, Botvinick M (2009). Machine learning classifiers and fMRI: a tutorial overview. NeuroImage.

[33] Kotsiantis SB. Supervised machine learning: a review of classification techniques. Informatica. 2007;31:249–68.

[34] Kuhn M. Caret package. Journal of Statistical Software. 2008;28(5):1–26.

[35] Steven MK (1988). Modern spectral estimation: theory and application.

[36] van Putten MJ (2004). A brain symmetry index (BSI) for online EEG monitoring in carotid endarterectomy. Clin Neurophysiol.

[37] van Putten MJ (2007). The revised brain symmetry index. Clin Neurophysiol.

[38] de Vos CC (2008). Continuous EEG monitoring during thrombolysis in acute hemispheric stroke patients using the brain symmetry index. J Clin Neurophysiol.

[39] van Putten MJ, Tavy DL (2004). Continuous quantitative EEG monitoring in hemispheric stroke patients using the brain symmetry index. Stroke.

[40] Cursi M (2005). Electroencephalographic background desynchronization during cerebral blood flow reduction. Clin Neurophysiol.

[41] Noirhomme Q (2014). Automated analysis of background EEG and reactivity during therapeutic hypothermia in comatose patients after cardiac arrest. Clin EEG Neurosci.

[42] Hermans MC (2016). Quantification of EEG reactivity in comatose patients. Clin Neurophysiol.

[43] Sitt JD (2014). Large scale screening of neural signatures of consciousness in patients in a vegetative or minimally conscious state. Brain.

[44] Chennu S (2014). Spectral signatures of reorganised brain networks in disorders of consciousness. PLoS Comput Biol.

[45] Noirhomme Q, Brecheisen R, Lesenfants D, Antonopoulos G, Laureys S. “Look at my classifier’s result”: disentangling unresponsive from (minimally) conscious patients. NeuroImage. 2017;145:288–303. 10.1016/j.neuroimage.2015.12.006 26690804

[46] Salti M, et al. Distinct cortical codes and temporal dynamics for conscious and unconscious percepts. elife. 2015;4. 10.7554/eLife.05652 PMC4467230 25997100

[47] Zubler F (2015). Prognostic and diagnostic value of EEG signal coupling measures in coma.

[48] Wijdicks EF (2015). Comparison of the full outline of UnResponsiveness score and the Glasgow coma scale in predicting mortality in critically ill patients*. Crit Care Med.

[49] Giacino JT, Kalmar K, Whyte J (2004). The JFK coma recovery scale-revised: measurement characteristics and diagnostic utility. Arch Phys Med Rehabil.

[50] Parthasarathy S, Tobin MJ (2004). Sleep in the intensive care unit. Intensive Care Med.

[51] Cologan V (2010). Sleep in disorders of consciousness. Sleep Med Rev.

[52] Kuhn M (2008). Building predictive models in R using the caret package. J Stat Softw.

[53] Temko A (2011). EEG-based neonatal seizure detection with support vector machines. Clin Neurophysiol.

[54] Greene BR (2008). Classifier models and architectures for EEG-based neonatal seizure detection. Physiol Meas.

[55] Nagaraj SB (2014). Robustness of time frequency distribution based features for automated neonatal EEG seizure detection. Conf Proc IEEE Eng Med Biol Soc.

[56] Samiee K, Kovacs P, Gabbouj M (2015). Epileptic seizure classification of EEG time-series using rational discrete short-time fourier transform. IEEE Trans Biomed Eng.

[57] Oweis RJ, Abdulhay EW (2011). Seizure classification in EEG signals utilizing Hilbert-Huang transform. Biomed Eng Online.

[58] Lee SH (2014). Classification of normal and epileptic seizure EEG signals using wavelet transform, phase-space reconstruction, and Euclidean distance. Comput Methods Prog Biomed.

[59] Chiang CY (2011). Seizure prediction based on classification of EEG synchronization patterns with on-line retraining and post-processing scheme. Conf Proc IEEE Eng Med Biol Soc.

[60] Bajaj V, Pachori RB (2012). Classification of seizure and non-seizure EEG signals using empirical mode decomposition. IEEE Trans Inf Technol Biomed.

[61] Muller KR (2008). Machine learning for real-time single-trial EEG-analysis: from brain-computer interfacing to mental state monitoring. J Neurosci Methods.

[62] Olofsen E, Sleigh J, Dahan A (2008). Permutation entropy of the electroencephalogram: a measure of anaesthetic drug effect. Br J Anaesth.

