# Supplementary material for: EEG dynamical correlates of focal and diffuse causes of coma
Source: BMC Neurol. 2017 Nov 15;17:197. doi: 10.1186/s12883-017-0977-0 (PMC5688694; doi:10.1186/s12883-017-0977-0)
Supplement: Additional file 1: Table S1. — Additional details regarding study population. (DOCX 12 kb) [file 12883_2017_977_MOESM1_ESM.docx]

Additional file 1 Table S1

| **Patient number** | **Clinically-Adjudicated Primary DLOC Etiology** | **GCS** | **Focal/Diffuse** |
| --- | --- | --- | --- |
| 1 | Subarachnoid hemorrhage with diffuse cerebral edema | 7 | Diffuse |
| 2 | Posterior fossa AVM rupture and brainstem compression | 7 | Focal |
| 3 | Frontal intraparenchymal hemorrhage from aneurysmal bleeding | 3 | Focal |
| 4 | Leptomeningeal spread of cancer with diffuse edema | 3 | Diffuse |
| 5 | Left-sided tumor and edema | 3 | Focal |
| 6 | Cryptococcal meningitis | 7 | Diffuse |
| 7 | Hypoxic-ischemic injury | 7 | Diffuse |
| 8 | Cerebellar hemorrhage compressing brainstem | 8 | Focal |
| 9 | Right-sided intraparenchymal hemorrhage from aneurysmal bleeding | 3 | Focal |
| 10 | Left-sided intraparenchymal hemorrhage | 4 | Focal |
| 11 | Right-sided intraparenchymal hemorrhage | 7 | Focal |
| 12 | Left subdural hematoma | 6 | Focal |
| 13 | Brainstem ischemia | 4 | Focal |
| 14 | Diffuse TBI | 3 | Diffuse |
| 15 | Cardiac arrest | 4 | Diffuse |
| 16 | Left-sided stroke with hemorrhagic conversion | 7 | Focal |
| 17 | Brainstem bleeding from AVM | 6 | Focal |
| 18 | Hydrocephalus | 6 | Diffuse |
| 19 | Cardiac arrest | 3 | Diffuse |
| 20 | Diffuse ischemic injury | 3 | Diffuse |
| 21 | Diffuse ischemic injury | 7 | Diffuse |
| 22 | Brainstem hemorrhage and ischemia | 6 | Focal |
| 23 | Diffuse cerebral edema and subarachnoid hemorrhage | 7 | Diffuse |
| 24 | Hydrocephalus | 4 | Diffuse |
| 25 | Left-sided stroke with hemorrhagic conversion | 5 | Focal |
| 26 | Uremia | 8 | Diffuse |
| 27 | Cardiac arrest | 2T | Diffuse |
| 28 | Pneumococcal meningitis | 6 | Diffuse |
| 29 | Left-sided intraparenchymal hemorrhage | 9 | Focal |
| 30 | Left-sided intraparenchymal hemorrhage | 8 | Focal |
| 31 | Left-sided intraparenchymal hemorrhage | 9 | Focal |
| 32 | Right-sided subdural hemorrhage | 8 | Focal |
| 33 | Right-sided stroke | 5 | Focal |
| 34 | Diffuse TBI | 8 | Diffuse |
| 35 | Brainstem compression | 6 | Focal |
| 36 | Left-sided intraparenchymal hemorrhage | 6 | Focal |
| 37 | Brainstem compression | 4 | Focal |
| 38 | Hydrocephalus and diffuse subarachnoid hemorrhage | 9 | Diffuse |
| 39 | Viral meningitis | 8 | Diffuse |
| 40 | Hydrocephalus and diffuse subarachnoid hemorrhage | 7 | Diffuse |
